# *In Situ* Visualization of the pKM101-Encoded Type IV Secretion System Reveals a Highly Symmetric ATPase Energy Center

**DOI:** 10.1128/mBio.02465-21

**Published:** 2021-10-12

**Authors:** Pratick Khara, Liqiang Song, Peter J. Christie, Bo Hu

**Affiliations:** a Department of Microbiology and Molecular Genetics, McGovern Medical School, Houston, Texas, USA; Washington University School of Medicine; Washington University School of Medicine

**Keywords:** cryo-electron tomography, DNA conjugation, type IV secretion, pilus, protein transport, traffic ATPase, protein translocation

## Abstract

Bacterial conjugation systems are members of the type IV secretion system (T4SS) superfamily. T4SSs can be classified as “minimized” or “expanded” based on whether they are composed of a core set of signature subunits or additional system-specific components. Prototypical minimized systems mediating Agrobacterium tumefaciens transfer DNA (T-DNA) and pKM101 and R388 plasmid transfer are built from subunits generically named VirB1 to VirB11 and VirD4. We visualized the pKM101-encoded T4SS in its native cellular context by *in situ* cryo-electron tomography (CryoET). The T4SS_pKM101_ is composed of an outer membrane core complex (OMCC) connected by a thin stalk to an inner membrane complex (IMC). The OMCC exhibits 14-fold symmetry and resembles that of the T4SS_R388_ analyzed previously by single-particle electron microscopy. The IMC is highly symmetrical and exhibits 6-fold symmetry. It is dominated by a hexameric collar in the periplasm and a cytoplasmic complex composed of a hexamer of dimers of the VirB4-like TraB ATPase. The IMC closely resembles equivalent regions of three expanded T4SSs previously visualized by *in situ* CryoET but differs strikingly from the IMC of the purified T4SS_R388_, whose cytoplasmic complex instead presents as two side-by-side VirB4 hexamers. Analyses of mutant machines lacking each of the three ATPases required for T4SS_pKM101_ function supplied evidence that TraB_B4_ as well as VirB11-like TraG contribute to distinct stages of machine assembly. We propose that the VirB4-like ATPases, configured as hexamers of dimers at the T4SS entrance, orchestrate IMC assembly and recruitment of the spatially dynamic VirB11 and VirD4 ATPases to activate the T4SS for substrate transfer.

## INTRODUCTION

Many species of bacteria deploy type IV secretion systems (T4SSs) to deliver DNA or protein substrates to target cells (1–3). T4SSs designated “minimized” systems are assembled from a core set of signature subunits, while others termed “expanded” are compositionally and structurally more complex, possibly reflecting adaptations arising over evolutionary time for specialized functions (3). In Gram-negative species, minimized systems are assembled from ∼12 subunits named VirB1 to VirB11 (VirB1-VirB11) and VirD4 based on the paradigmatic Agrobacterium tumefaciens VirB/VirD4 T4SS (3). Three subunits (VirB7, VirB9, and the C terminus of VirB10) assemble as an outer membrane (OM) core complex (OMCC) that spans the distal region of the periplasm and OM (4). Four integral membrane components (VirB3, VirB6, VirB8, the N terminus of VirB10), and two or three ATPases (VirB4 and VirD4 with or without VirB11) together comprise the inner membrane (IM) complex (IMC) (5, 6). Some T4SSs elaborate an extracellular organelle termed the conjugative pilus from homologs of the VirB2 pilin and VirB5 pilus tip subunit (3). Expanded systems are composed of homologs or orthologs of most or all of the VirB/VirD4 subunits plus as many as 20 components that are system specific (3).

To better understand the mechanism of action of T4SSs and the structural bases underlying the functional diversity of this translocation superfamily, there is growing interest in solving the structures of intact machines and machine subassemblies. OMCCs are generally stable and amenable to purification, and structures are now available for OMCCs from several minimized and expanded systems at resolutions approaching ∼3 Å (4, 6–12). Structural analyses of IM portions of T4SSs have been considerably more challenging due to problems of instability and dissociation during purification. Presently, one structure exists for a minimized system encoded by the conjugative plasmid R388. Designated the VirB_3-10_ complex, this structure was obtained by the overproduction of the VirB3-VirB10 homologs, affinity purification of the detergent-solubilized complex, and analysis by negative-stain electron microscopy (nsEM) (6). The VirB_3-10_ complex consists of the OMCC and IMC connected by a thin, flexible stalk. The IMC is composed of a highly asymmetric IM platform connected to two side-by-side hexamers of the VirB4 ATPase extending into the cytoplasm. In an updated structure, two dimers of the VirD4 ATPase were shown to integrate between the VirB4 barrels (13).

IMCs of expanded T4SSs have not yet been analyzed by single-particle EM. However, recent advances using *in situ* cryo-electron tomography (CryoET) have enabled the visualization of the Legionella pneumophila Dot/Icm, Helicobacter pylori Cag, and F plasmid-encoded Tra T4SSs (designated T4SS_Dot/Icm_, etc., here) in the native context of the cell envelope (14–21). Remarkably, in contrast to the IMC of the VirB_3-10_ structure, the IMCs of all three expanded systems clearly exhibit 6-fold symmetry, and the VirB4 ATPases assemble as a central hexamer of dimers at the channel entrance (16–18).

Here, we solved the *in situ* structure of the pKM101-encoded T4SS, which is phylogenetically (see Fig. S1A in the supplemental material) and functionally closely related to the R388-encoded T4SS, to the extent that the two minimized systems can translocate each other’s plasmids, and some machine subunits are exchangeable (8). We report that the IMC of the *in situ* T4SS_pKM101_ adopts the 6-fold symmetry observed for the equivalent regions of the expanded T4SSs. Most strikingly, the VirB4 homolog TraB (TraB_B4_) is arranged as a central hexamer of dimers, not the side-by-side hexameric barrels visualized for this ATPase in the purified VirB_3-10_ complex. Mutant machines lacking TraB_B4_ or VirB11-like TraG exhibit structural differences in the IMC compared with the wild-type (WT) machine, which is suggestive of contributions of these ATPases to distinct stages of T4SS_pKM101_ machine assembly. Together, our findings support a model in which the VirB4 ATPases, configured as central hexamers of dimers at the bases of T4SSs, play key roles in several early-stage morphogenetic reactions required for machine biogenesis.

10.1128/mBio.02465-21.2 FIG S1 Genes and encoded functions of “minimized” type IV secretion systems (T4SSs) in Gram-negative species. Download FIG S1, PDF file, 1.1 MB. Copyright © 2021 Khara et al. 2021 Khara et al. https://creativecommons.org/licenses/by/4.0/ This content is distributed under the terms of the Creative Commons Attribution 4.0 International license.

## RESULTS AND DISCUSSION

### *In situ* detection of the pKM101 nanomachine.

**FIG 1:**
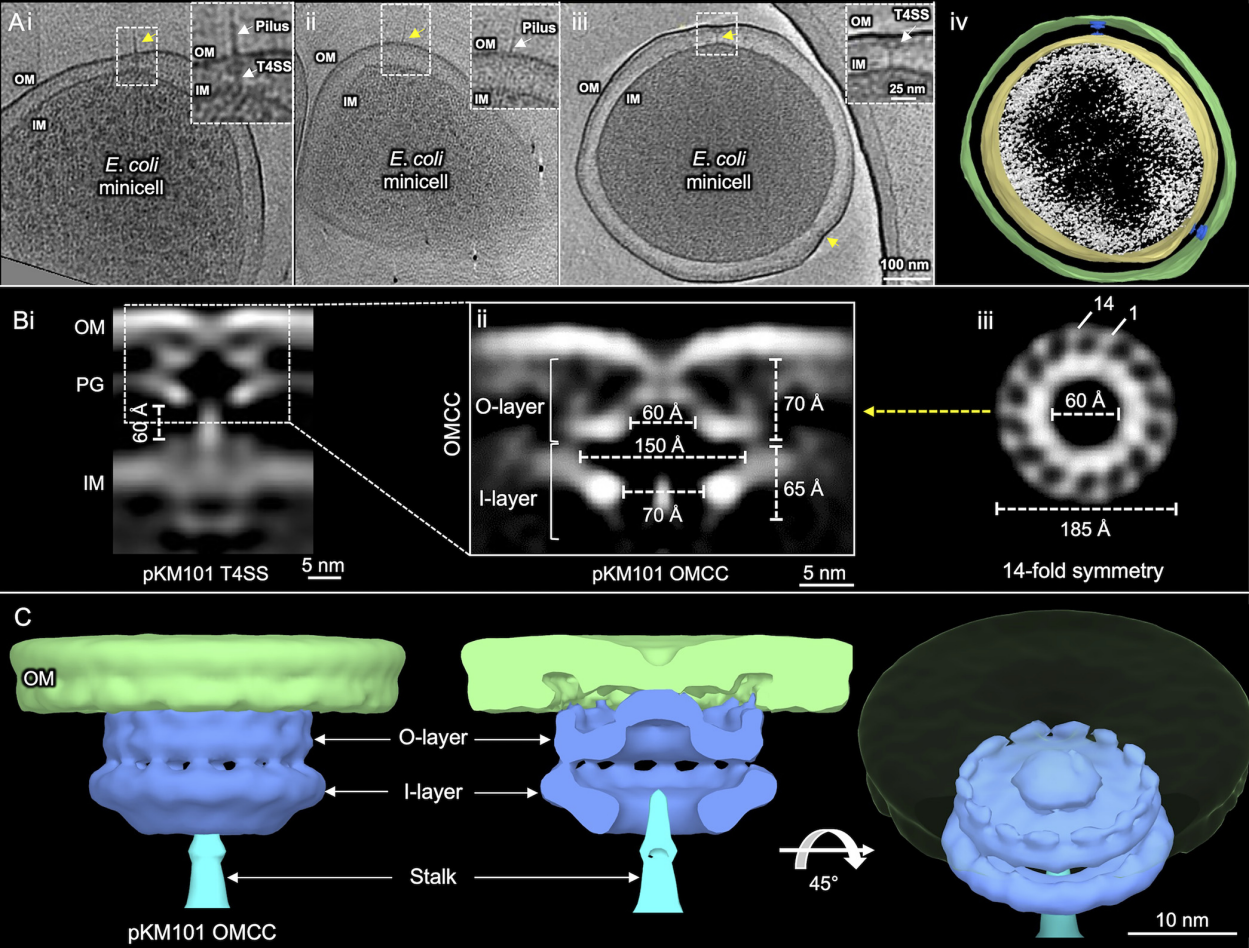
E. coli minicells carrying the pKM101-encoded type IV secretion system (T4SS_pKM101_) and *in situ* structure of the outer membrane core complex (OMCC) of the T4SS_pKM101_ revealed by CryoET and subtomogram averaging. (Ai to iii) Tomographic slices from representative E. coli minicells showing T4SSs embedded between the outer membrane (OM) and the inner membrane (IM). pKM101 pili were associated with a few visualized T4SSs, although pilus-associated OM structures were without any periplasmic densities. The T4SS and novel structures are marked with yellow arrows. The boxed regions are magnified to show the T4SS with and without the associated pilus and also pilus-associated OM structures. (iv) 3D surface view of the E. coli minicell in panel Aiii showing T4SSs. (Bi) Central slice of the averaged structure of the T4SS in the cell envelope. (ii) After refinement, details of the OMCC are visible. The widths and heights of O-layer and I-layer chambers are shown. (iii) Cross-sectional view of the region in panel Bii marked by a yellow arrow showing 14-fold symmetry of the OMCC. (C) 3D surface renderings of the OMCC shown in different views.

To visualize T4SS_pKM101_ nanomachines, we deployed an Escherichia coli *mreB minC* mutant carrying pKM101 to generate small (<300 nm in diameter) minicells (17). Minicells are ideal for *in situ* CryoET because of their small size and full metabolic capacity (22), including the ability to deliver plasmids such as F (17) or pKM101 (see Fig. S1B in the supplemental material) through encoded T4SSs to recipient cells. We used a high-throughput CryoET pipeline to visualize thousands of E. coli minicells (see Fig. S2 for the workflow). The pKM101 nanomachines were smaller and more difficult to detect than the F plasmid-encoded T4SS or other expanded systems that we have previously characterized (16–18), but we were able to detect pKM101-encoded structures among every 2 or 3 minicells examined (Fig. 1Ai to iv; Movie S1). Importantly, minicell preparations from the parental strain UU2834 alone lack these surface structures, confirming that the presence of pKM101 in the host strain is required for their elaboration.

10.1128/mBio.02465-21.3 FIG S2 Workflow for *in situ* CryoET. Download FIG S2, PDF file, 0.5 MB. Copyright © 2021 Khara et al. 2021 Khara et al. https://creativecommons.org/licenses/by/4.0/ This content is distributed under the terms of the Creative Commons Attribution 4.0 International license.

10.1128/mBio.02465-21.8 MOVIE S1 3D visualization of a tomographic reconstruction and the T4SS_pKM101_ in E. coli minicells. Download Movie S1, MOV file, 11.0 MB. Copyright © 2021 Khara et al. 2021 Khara et al. https://creativecommons.org/licenses/by/4.0/ This content is distributed under the terms of the Creative Commons Attribution 4.0 International license.

The pKM101-encoded structures consist of periplasmic cone-shaped complexes near the outer membrane (OM) without or with associated thin “stalk” structures extending to the IM (Fig. 1Ai and iii). OMCCs lacking stalk structures represented about half of the initially picked particles but likely represent assembly intermediates or dead-end complexes and were not examined further (Fig. S2). The T4SS_pKM101_ also elaborates brittle pili that are readily detached or sloughed from cells (23). We detected some pKM101-encoded pili, bound either to the OMCC-stalk structures or to sites on the OM devoid of underlying basal densities (Fig. 1Ai and ii; Fig. S3). Because the pKM101 pili were rarely detected, we focused on solving the *in situ* structure of the cell-envelope-spanning nanomachine to allow comparisons with other T4SS structures solved *in situ* or *in vitro* (4, 6, 7).

10.1128/mBio.02465-21.4 FIG S3 Detection of a pKM101 pilus docked on the E. coli outer membrane. Download FIG S3, PDF file, 1.7 MB. Copyright © 2021 Khara et al. 2021 Khara et al. https://creativecommons.org/licenses/by/4.0/ This content is distributed under the terms of the Creative Commons Attribution 4.0 International license.

### Visualization of the *in situ* OMCC.

From 287 nanomachine subtomograms extracted from 560 tomographic reconstructions, we generated an *in situ* structure of the OMCC at a resolution of ∼37 Å (Fig. 1B; Fig. S2). Three-dimensional (3D) classifications revealed 14-fold symmetrical features of the OMCC, which were resolved further by imposing 14-fold symmetry during refinement (Fig. S2). In the refined structure, the OMCC is clearly seen attached to the OM, where it causes an invagination of the outer leaflet (Fig. 1Bi and ii). The upper region, designated the O-layer (4), is 185 Å wide and 70 Å in height. In the side view, the complex forms at least two contacts with the OM, the first mediated by a central cap and the second mediated by the periphery of the OMCC (Fig. 1Bii). In the middle of the central cap and extending across the OM is a region of lower density that might correspond to the OM-spanning channel. In the top-down view, the periphery of the OMCC clearly consists of 14 knobs arranged in a ring of ∼185 Å in width (Fig. 1Biii). The knobs are connected via spokes to a central contiguous ring that conforms to the base of the cap. In 3D renderings, it is evident that the 14 peripheral knobs interact with the OM (Fig. 1C). Notably, besides the invagination of the OM at the cap junction, the region of the OM between the central cap and peripheral contacts lacks an inner leaflet, suggesting that the OM undergoes extensive remodeling during machine biogenesis (Fig. 1Bii and Fig. 1C).

The O-layer chamber is closed at the OM junction and widens to ∼150 Å where it joins the lower region of the OMCC known as the I-layer (4). The I-layer has a height of 65 Å and is slightly wider than the O-layer, although the outer boundary of the O-layer is blurred because of the density contributed by the peptidoglycan (PG) layer (Fig. 1Bii). The I-layer narrows at its base, where the central chamber has a diameter of ∼70 Å. A stalk density embeds into the central cavity and projects through the periplasm to the IM (Fig. 1Bi and ii and Fig. 1C). Overall, the *in situ* OMCC structure has a total height of 135 Å (Fig. 1Bii).

Although the resolution of the visualized OMCC (∼37 Å) is lower than that achieved by single-particle analyses (<20 Å) (4, 24), the *in situ* and *in vitro* OMCCs exhibit 14-fold symmetry and have similar cross-section dimensions of 185 Å (Fig. S4). They are also composed of distinct O- and I-layers that house large central chambers (4, 7). The *in vitro* structure, however, is more elongated (∼185 Å in height) than the *in situ* structure (135 Å) (Fig. S4B and C). The O-layer and the upper portion of the I-layer are intrinsically stable due to extensive networks of interactions between the TraF_B10_ and TraO_B9_ constituents (24). In contrast, the lower portion of the I-layer, which is built from α-helical linker domains of TraF_B10_ that connect the OMCC to the IM, is highly flexible (4, 24). Therefore, we suspect that the region of the OMCC that resolves well in the *in situ* structure corresponds to the O-layer and the upper portion of the I-layer, whereas the linker domains comprising the lower portion of the I-layer either are too flexible for detection *in vivo* or fold inward to form part of the central stalk.

10.1128/mBio.02465-21.5 FIG S4 Comparisons of the outer membrane core complexes (OMCCs) and stalks/channels of “minimized” and “expanded” T4SSs. Download FIG S4, PDF file, 1.1 MB. Copyright © 2021 Khara et al. 2021 Khara et al. https://creativecommons.org/licenses/by/4.0/ This content is distributed under the terms of the Creative Commons Attribution 4.0 International license.

Gratifyingly, an X-ray structure of the O-layer (7) fits well into the O-layer of the *in situ* structure (Fig. S4D). The OMCC of the *in vitro* VirB_3-10_ complex also generally superimposes well onto the equivalent subassembly of the *in situ* T4SS_pKM101_, although the latter structure has additional densities at the top comprising the peripheral OM contacts and laterally that might correspond to the associated PG (Fig. S4Ei to iii). The convergence of OMCC architectures from the R388 and pKM101 systems is in line with previous findings that the OMCC from the R388 machine can be swapped for that of the pKM101 system to yield a functional chimeric system (8).

Although a flexible stalk connecting the OMCC and the inner membrane complex (IMC) was previously visualized in the VirB_3-10_ complex (6), at the time, it was not known if the stalk corresponded to a central channel that was structurally distorted during detergent solubilization of the nanomachine (Fig. S4Ei and iii). Here, our finding that a stalk density (Fig. S4Eiii) lacking a discernible channel joins the OMCC to the IMC in the *in situ* T4SS_pKM101_ confirms that the stalk is a prominent feature of minimized systems. This distinguishes the minimized systems from expanded systems such as the F plasmid Tra and L. pneumophila Dot/Icm T4SSs (16, 17), whose *in situ* structures clearly possess central channels bridging the IMC and OMCC subassemblies (Fig. S4Ei to iii). It is also interesting that the central stalk of the VirB_3-10_ structure spans a gap of ∼33 Å between the OMCC and the IMC. Here, however, we observed that the gap between the OMCCs and IMCs of different T4SS_pKM101_ machines was more variable and in the range of ∼50 to 70 Å. *In situ* CryoET captures structural snapshots of dynamic processes; this variability might reflect the ensemble of the T4SS_pKM101_ visualized at different stages of assembly or activation.

### Visualization of the *in situ* IMC.

**FIG 2:**
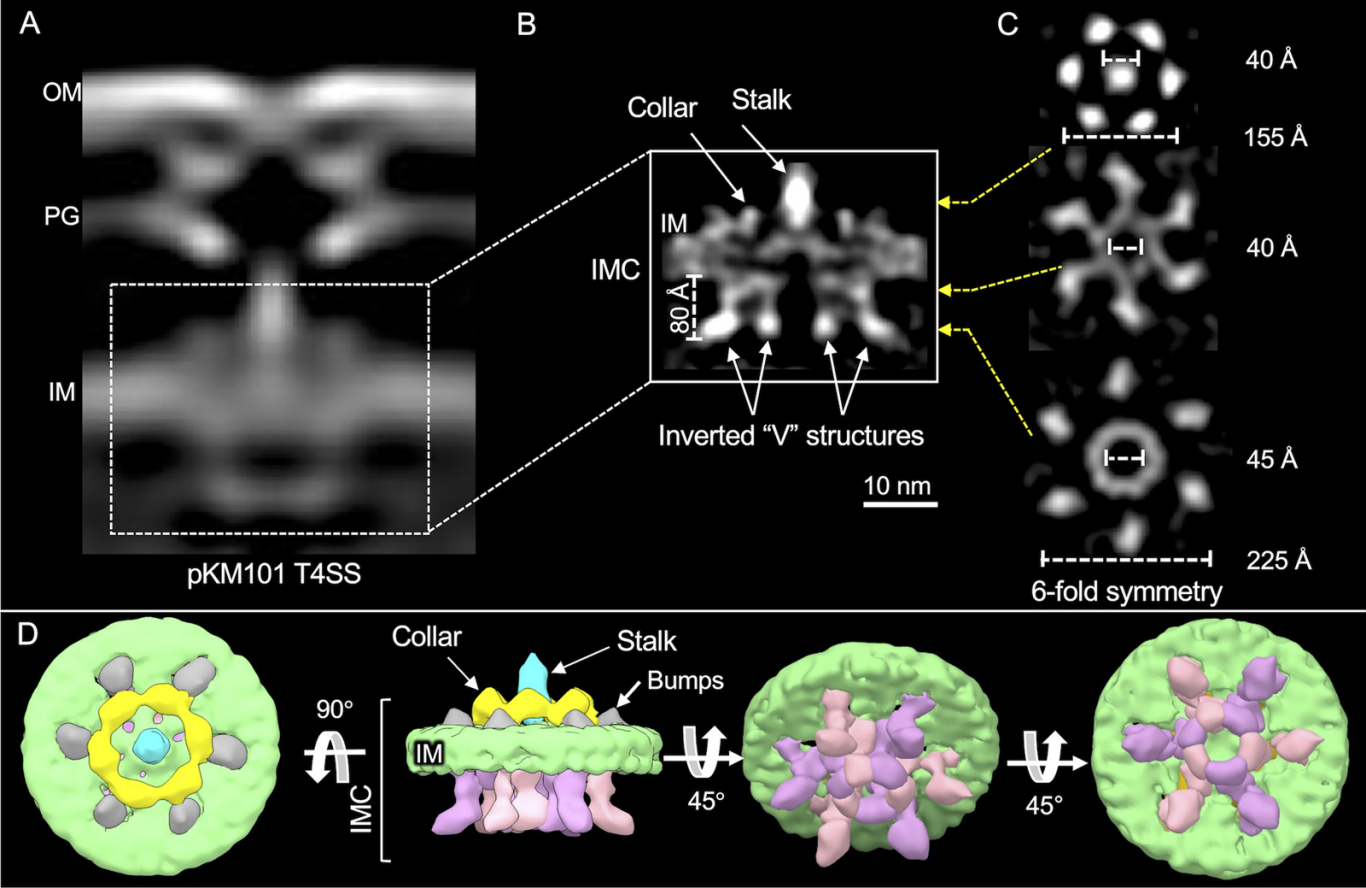
*In situ* structure of the inner membrane complex (IMC) of the pKM101-encoded type IV secretion system (T4SS_pKM101_) revealed by CryoET and subtomogram averaging. (A) Central slice of the averaged structure of the T4SS in the cell envelope. (B) After refinement, details of the IMC are visible. The stalk, collar, and inverted V structures along with the height of its arms are shown. (C) Cross-sectional views of the regions in panel B marked by yellow arrows showing 6-fold symmetry of the IMC. The collar exists as a hexameric ring-like structure around the central stalk. (D) 3D surface renderings of the IMC shown in different views.

Next, we refined the structure of the IMC using class averages of machines with detectable OMCC and IMC subassemblies (Fig. 2A and B; Fig. S2). Notable features of the IMC include a distinct collar surrounding the central stalk, which in the top-down view presents as six knobs arranged in a ring of 155 Å. The collar was flanked by six protrusions or “bumps” that were clearly distinct from the IM density (Fig. 2B and D). At the cytoplasmic face of the IM, the IMC was dominated by two side-by-side inverted “V” structures with apices embedded in the IM and “arms” projecting ∼80 Å into the cytoplasm (Fig. 2B). In the end-on view, six V structures clearly form two concentric circles, the outer arms of the V’s were configured as a knobbed ring of ∼225 Å in diameter, and the inner arms joined together as a central hexameric ring with an outer diameter of ∼60 Å and a lumen of ∼45 Å. As for the periplasmic collar, the 6-fold symmetry of the concentric rings was readily visible among the class-average images without symmetry imposed (Fig. S2). The structure was resolved further by imposing 6-fold symmetry during refinement (Fig. 2B to D; Fig. S2).

**FIG 3:**
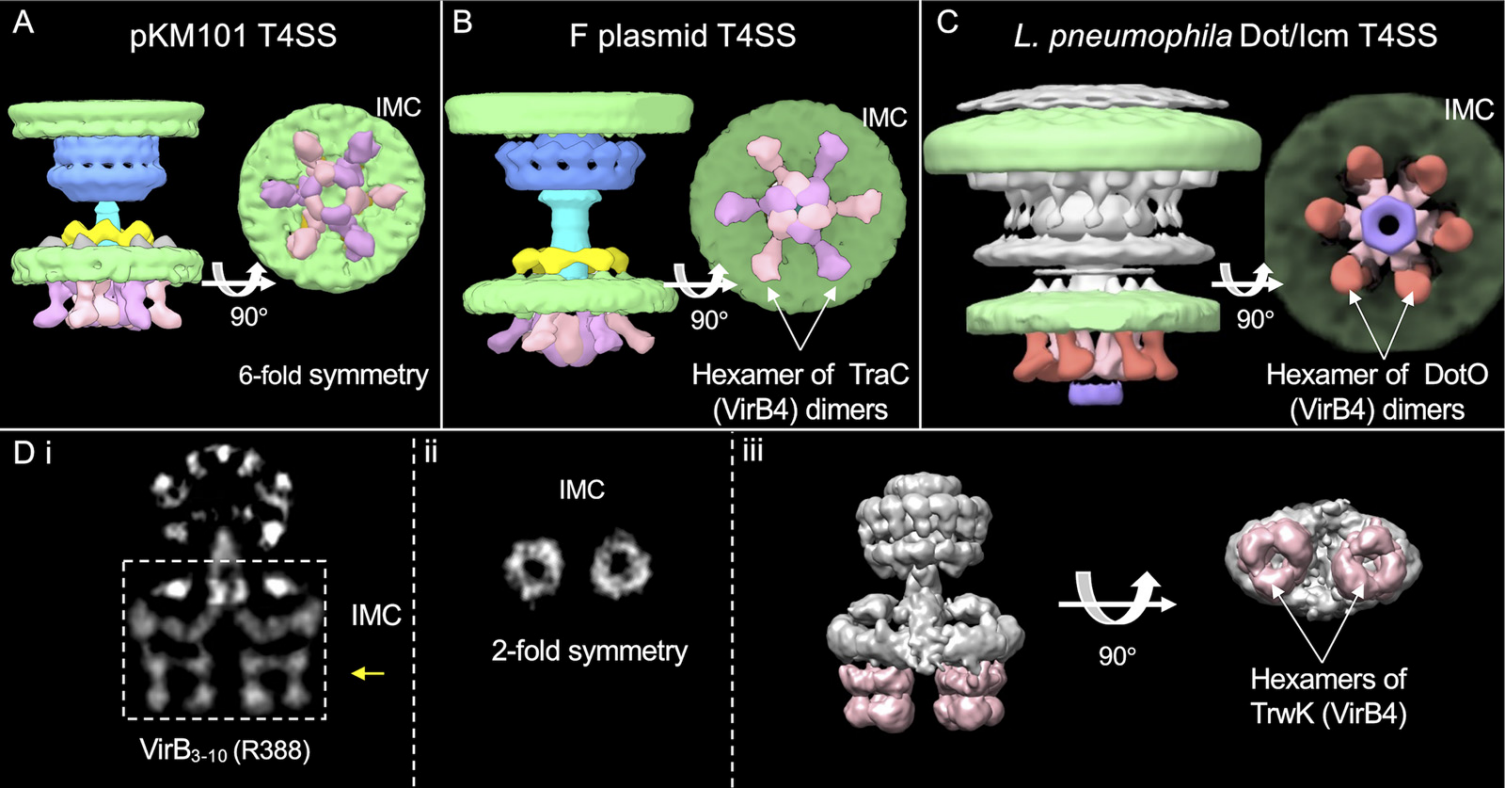
Comparison of the inner membrane complexes (IMCs) solved by CryoET and single-particle analysis. (A to C) Comparison of the CryoET-solved IMCs of type IV secretion systems encoded by pKM101 (T4SS_pKM101_), the F plasmid (T4SS_pED208_), and L. pneumophila (T4SS_Dot/Icm_). 3D surface renderings show 6-fold symmetric IMCs marked by the hexamer-of-dimer arrangement of VirB_4_ homologs. (Di) Central slice of the averaged structure of the purified VirB_3-10_ substructure encoded by plasmid R388. (ii) Cross-sectional view of the region in panel Di marked by a yellow arrow showing that 2-fold symmetry exists in the IMC of purified VirB_3-10_. (iii) Surface rendering of the VirB_3-10_ substructure highlighting the IMC with two side-by-side hexamers of the TrwK/VirB4 ATPase (pink shading) (F plasmid EMDB identifiers EMD-9344 and EMD-9347, Dot/Icm EMDB identifiers EMD-7611 and EMD-7612, and VirB_3-10_ EMDB identifier EMD-2567). All EMD structures can be accessed through the EMDB URL followed by the identifier listed, e.g., https://www.ebi.ac.uk/emdb/EMD-9344.

The structure of the pKM101 IMC bears striking similarities to IMCs associated with the F plasmid-encoded Tra and L. pneumophila Dot/Icm systems, whose structures were also solved by *in situ* CryoET (Fig. 3A to C) (16, 17). Most notably, the cytoplasmic complexes of all three systems appear as side-by-side V’s in the side view and as outer knobbed and inner continuous rings of similar sizes in the end-on view. In studies of the F and Dot/Icm machines, structural analyses of mutant machines deleted of each of the T4SS ATPases, coupled with density tracing of a green fluorescent protein (GFP) moiety fused to a VirB4 homolog, established that the V structures correspond to dimers of VirB4-like ATPases (16, 17). The cytoplasmic complexes of the F plasmid and Dot/Icm systems therefore consist primarily of VirB4 subunits arranged as a central hexamer of dimers at the base of the translocation channel. The IMC of the H. pylori Cag T4SS is architecturally more complex than the IMCs of the F plasmid-encoded and Dot/Icm systems, yet VirB4-like Cagβ is similarly configured as a hexamer of dimers at the Cag channel entrance (18).

The pKM101 IMC visualized here differs remarkably from that associated with the *in vitro* VirB_3-10_ structure (Fig. 2B and C and Fig. 3A and Di to iii) (6). Most notably, the pKM101 IMC is highly symmetric in its overall 6-fold symmetrical periplasmic collar and cytoplasmic complex. The VirB_3-10_ IMC is asymmetric and dominated by side-by-side barrel complexes, which consist at least partly of the VirB4 homolog TrwK, as shown by gold labeling (6). A hexameric arrangement for the two barrels was inferred by previous findings that TrwK_B4_ assembles *in vitro* as a homohexamer and results of stoichiometric analyses showing that the VirB_3-10_ complex is composed of 12 copies of TrwK_B4_ (6, 25).

### The pKM101 cytoplasmic complex is dominated by VirB4-like TraB.

**FIG 4:**
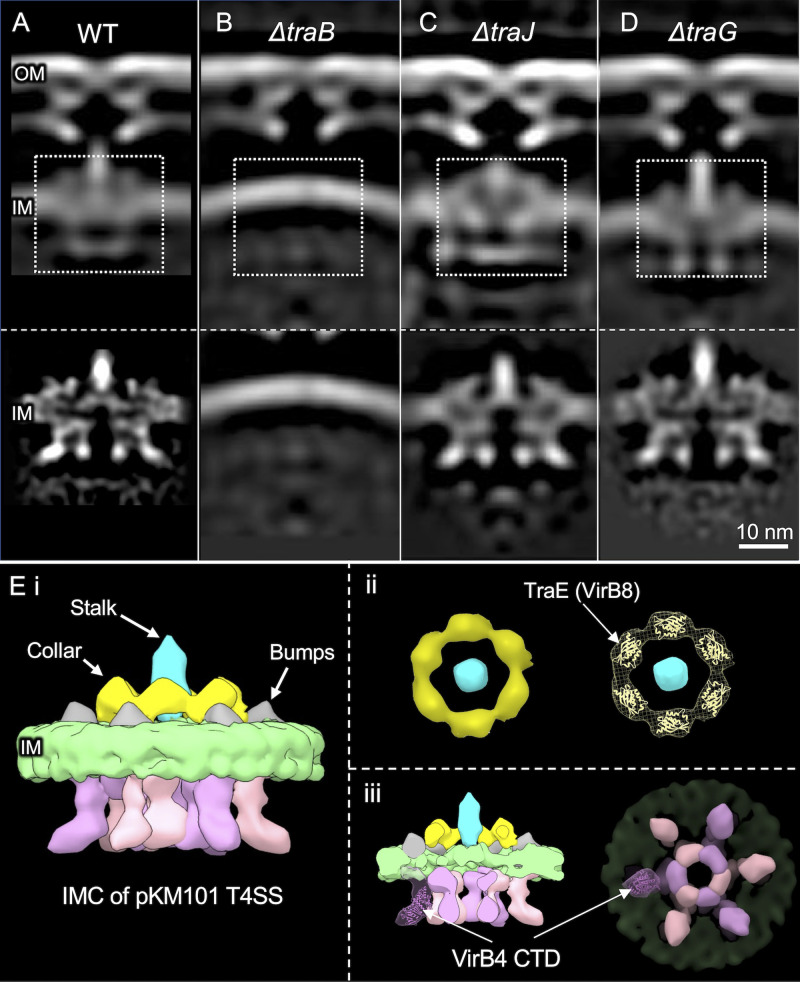
Architecture of T4SS_pKM101_ mutant machines from strains lacking one of the Tra ATPases and comparison of the IMC from T4SS_pKM101_ with those from other solved structures. (A to D, top) Central slices of the averaged structures from strains carrying native pKM101 or the Δ*traB*, Δ*traJ*, and Δ*traG* mutant plasmids. (Bottom) Refined IMCs of the corresponding strains. (Ei) 3D surface rendering of the IMC of the T4SS_pKM101_. (ii) End-on view of the hexameric collar. A crystal structure of the periplasmic domain of pKM101-encoded TraE_B8_ (PDB accession number 5I97; https://www.rcsb.org/structure/5I97) fits well in the lobe-like structure of the collar. (iii) The crystal structure of the C-terminal domain (CTD) of VirB4 ATPase from Thermoanaerobacter pseudethanolicus (PDB accession number 4AG5; https://www.rcsb.org/structure/4AG5) fits well in one of the arms of the TraB hexamer of dimers.

To define the contributions of VirB4-like TraB to the T4SS_pKM101_, we imaged Δ*traB_B4_* mutant machines (100 machines from 254 tomographic reconstructions) (Fig. S5). We previously reported that *traB_B4_* expression in *trans* fully complements a Δ*traB_B4_* mutation, confirming that the mutation is nonpolar on downstream gene expression (8). Subvolume class averages of the Δ*traB_B4_* mutant machines consisted of the OMCC without associated IMC densities (Fig. S5). Most notably, the Δ*traB_B4_* machines lacked cytoplasmic densities dominated by the concentric hexameric rings (Fig. 4B; Fig. S5), indicating that TraB_B4_ adopts the same hexamer-of-dimer architectures observed for VirB4 homologs associated with the F plasmid-encoded, Dot/Icm, and Cag T4SSs (16–18). This architecture is compatible with the results of previous biochemical studies showing that TraB_B4_ and the closely related TrwK_B4_ from the R388 system purify as dimers or hexamers (25–28), as both of these oligomeric states are predicted from detergent extraction of a membrane ATPase with a hexamer-of-dimer configuration. The VirB4 ATPases are arranged so that their N-terminal domains (NTDs) associate tightly with the IM (28, 29), and the C-terminal domains (CTDs) consisting of RecA-like α/β structural folds extend into the cytoplasm (30, 31). An atomic structure of the CTD of a VirB4 homolog fitted optimally within densities comprising the proximal halves of the V arms (Fig. 4Eiii), which lends further support to the conclusion that visualized hexamer-of-dimer densities are composed of TraB_B4_.

10.1128/mBio.02465-21.6 FIG S5 Heterogeneity of the pKM101 T4SS machines revealed by class subvolume averaging. Download FIG S5, PDF file, 1.0 MB. Copyright © 2021 Khara et al. 2021 Khara et al. https://creativecommons.org/licenses/by/4.0/ This content is distributed under the terms of the Creative Commons Attribution 4.0 International license.

IMC densities other than the inverted V’s were also missing in Δ*traB_B4_* mutant machines, including the periplasmic stalk and surrounding collar and bumps (Fig. 4B; Fig. S5). VirB4 homologs associate peripherally with the IM (32) or at most possess small periplasmic domains (29), arguing against appreciable contributions of TraB_B4_ to the observed collar or stalk densities. However, several IMC subunits are strong candidates for constituting these densities. These include periplasmic linker domains of VirB10-like TraF and VirB5-like TraC, which are likely components of the stalk (6). VirB8-like TraE is a strong candidate for the central collar, as supported by a recent cryo-electron microscopy (CryoEM) structure showing that purified TraE_B8_ assembles as a homohexamer with dimensions matching those of the collar visualized *in situ* (33). Here, we also determined that an atomic structure of the periplasmic domain of TraE_B8_ (34) also fits well within each lobe of the *in situ* collar (Fig. 4Eii). Finally, like other VirB6 subunits (35), TraD_B6_ has a large central periplasmic domain that likely also contributes to one or more of the periplasmic densities. The absence of discernible periplasmic densities in the Δ*traB* mutant machines suggests that TraB_B4_-IMC subunit contacts are necessary for the stable assembly of the IMC. In line with this proposal, numerous studies have presented evidence that VirB4-like subunits form a network of stabilizing interactions with IMC constituents, including homologs of VirB3, VirB5, VirB8, and VirB10 (8, 36–39).

### Deletions of the TraJ or TraG ATPases do not detectably alter the *in situ* T4SS_pKM101_.

Nearly all T4SSs require a VirD4-like ATPase, which serves to recruit and deliver secretion substrates into the transfer channel. Designated type IV coupling proteins (T4CPs) or substrate receptors, VirD4 subunits are members of the SpoIIIE/FtsK superfamily of motor translocases (40, 41). To determine if VirD4-like TraJ contributes to densities of the T4SS_pKM101_, we imaged Δ*traJ_D4_* mutant machines (183 machines from 430 tomographic reconstructions). As observed with the WT machines, subvolume averaging yielded classes of Δ*traJ_D4_* mutant machines exhibiting only the OMCC or both the OMCC and IMC densities (Fig. S5). A refined structure generated from the latter classes showed no distortions compared to WT machines insofar as the OMCC, periplasmic collar and central stalk, and cytoplasmic V structures were clearly evident (Fig. 4C). TraJ_D4_ thus does not contribute detectably to the *in situ* T4SS_pKM101_ structure. The F plasmid-encoded Tra and L. pneumophila Dot/Icm machines were similarly unaltered upon the deletion of their respective VirD4 receptors (16, 17). In a recently updated *in vitro* structure of the R388-encoded T4SS, densities thought to correspond to one or two dimers of VirD4-like TrwB were shown to be integrated between the side-by-side VirB4 barrels (13). However, the *in situ* architecture of the Δ*traJ_D4_* mutant machines, together with evidence that VirD4-like subunits assemble as homohexamers (40, 42) and engage with T4SS channels only when activated by intracellular signals such as substrate binding and ATP hydrolysis (43–47), suggests that the *in vitro* VirB_3-10_/VirD4 complexes might represent transition-state structures.

Many T4SSs also require a third ATPase designated VirB11 for substrate transfer and pilus production (37, 48). VirB11 ATPases assemble as homohexamers that cofractionate with the cytoplasm and IM, the latter presumably in association with the T4SS (16, 49–51). To determine if TraG_B11_ contributes to the visualized IMC_pKM101_, we imaged Δ*traG_B11_* mutant machines (257 machines from 537 tomograms). We were unable to detect any density losses in the cytoplasmic complex of the Δ*traG* mutant machine compared with the WT machine (Fig. 4). This suggests that TraG_B11_ might associate dynamically with the T4SS_pKM101_, as shown previously for VirB11-like DotB in the L. pneumophila Dot/Icm system (16). In that system, the detection of DotB_B11_ at the base of DotO_B4_ required the deployment of a mutant form of DotB_B11_ capable of binding but not hydrolyzing ATP (16).

Interestingly, we also observed that the Δ*traG_B11_* mutant machines exhibited structural aberrations compared with the WT machines that were suggestive of profound effects of TraG_B11_ docking on the T4SS_pKM101_ channel architecture. Recall that OMCCs were associated with IMC densities in only ∼50% of the subvolume class averages of WT machines. In striking contrast, in Δ*traG_B11_* machines, ∼85% of visualized OMCCs were associated with IMC densities. Furthermore, the IMC densities were more clearly defined for the Δ*traG_B11_* mutant machines than for the WT machines, and notably, the central stalks were considerably elongated (Fig. 4; Fig. S5). These findings suggest that TraG_B11_ plays an important role in regulating the conformational status of the central stalk and IMC. Previous work has presented evidence that VirB11 functions as a switch to regulate pilus biogenesis versus DNA transport modes of action in the R388-encoded system (52). Furthermore, a recent *in situ* CryoET study documented structural changes in the IM upon the binding of DotB_B11_ to DotO_B4_ consistent with a role for this ATPase in the opening of the IM channel in the L. pneumophila Dot/Icm system (21). It is enticing to propose that TraG_B11_ binding with TraB_B4_ might similarly open the IM channel of the T4SS_pKM101_ and also induce structural transitions in the central stalk of importance for substrate passage to the cell exterior. Further studies examining the structural consequences of TraG_B11_ and TraJ_D4_ docking with the T4SS_pKM101_ are clearly warranted.

### Summary.

CryoET has emerged as a valuable complementary approach to single-particle CryoEM studies of bacterial secretion nanomachines (53). Although current resolutions achievable with CryoET are lower than those with CryoEM, structural definition of machines in their native contexts enables (i) validation of architectural features observed *in vitro*, (ii) assessments of machine structural variability within and between species, and (iii) visualization of dynamic aspects of machine biogenesis and function (3, 53, 54). Here, we present the first *in situ* structures of a minimized T4SS elaborated by the model conjugative plasmid pKM101. We showed that the *in vivo* T4SS_pKM101_ consists of two large substructures, the OMCC and the IMC, and that the former resembles structures of equivalent complexes solved *in vitro* (4, 7, 24). We further identified specific OM contacts, supplied evidence for OM remodeling during T4SS_pKM101_ biogenesis, and visualized a central stalk similar to that detected in the isolated VirB_3-10_ complex (6). We also gained evidence for contributions of TraG_B11_ to the assembly or configuration of the IMC and central stalk, in agreement with recent findings for DotB_B11_ in the Dot/Icm system (21). Most importantly, we show that TraB_B4_ assembles as a central hexamer of dimers, an oligomeric conformation similar to those of VirB4 homologs associated with the F-encoded Tra, L. pneumophila Dot/Icm, and H. pylori Cag T4SSs (16–18). IMCs of T4SSs are characteristically highly unstable and difficult to purify in the presence of detergents (4, 9–12, 24), raising the possibility that the side-by-side barrel arrangement described for TrwK_B4_ in the VirB_3-10_ complex (6) might be a structural artifact of machine purification.

**FIG 5:**
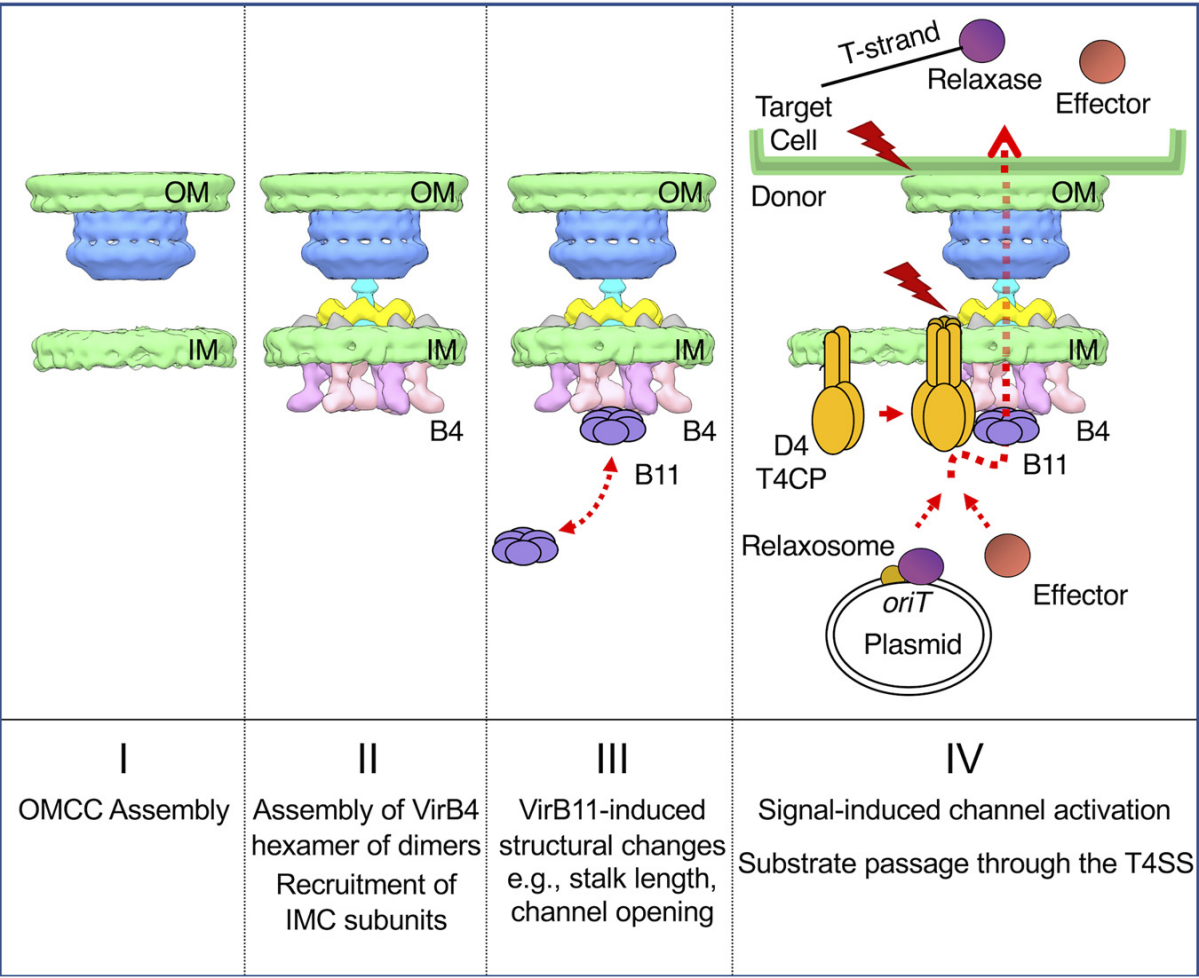
Working model depicting the contributions of the VirB4 ATPases to early stages of T4SS assembly and substrate routing. (Stage I) The OMCC assembles as an intrinsically stable substructure independently of contributions by the T4SS ATPases. (Stage II) VirB4 is recruited to the OMCC through contacts with the N-terminal cytoplasmic and IM transmembrane domains of VirB10. VirB4 assembles as a hexamer of dimers at the cytoplasmic face of the IM, where it recruits and stabilizes other IMC and stalk constituents. (Stage III) VirB4 serves as a docking site for the spatially dynamic VirB11 ATPase. Docked VirB11 regulates structural transitions necessary for channel activation. (Stage IV) The VirD4-like substrate receptor or T4CP (type IV coupling protein) oligomerizes and hydrolyzes ATP in response to the binding of DNA or protein substrates. The VirD4-substrate complex engages with and activates the T4SS channel. The ATPase energy center composed of the VirB4, VirB11, and VirD4 ATPases coordinates the processing and delivery of secretion substrates through the central lumen of VirB4 and into the translocation channel (37, 48). Red lightning bolts denote signals, such as substrate binding, VirD4 ATP hydrolysis, and the establishment of T4SS contact with the target cell, that activate the T4SS for transfer. Relaxase is the enzyme responsible for nicking the DNA strand (T-strand) destined for transfer; relaxosome is an assemblage of processing proteins at the origin-of-transfer (*oriT*) sequence that are responsible for nicking and unwinding the T-strand for transfer (see reference 2).

Together with previous biochemical and structural data (21, 36, 37, 45, 55), our findings support a model in which VirB4 ATPases play critical roles in several key steps of T4SS biogenesis, as depicted in Fig. 5. In stage I, the intrinsically stable OMCC assembles without contributions by VirB4 or other ATPases. This stage I reaction is supported by our *in situ* evidence that the pKM101 OMCC assembles in the absence of associated IMC densities (Fig. S2). In stage II, the OMCC recruits VirB4 through previously identified interactions between the ATPase and the cell-envelope-spanning VirB10 subunit (37). VirB4 then recruits or stabilizes other IMC components, including VirB3, VirB5, VirB6, and VirB8, to yield the IMC. This stage II reaction is supported by our findings that TraB_B4_ is required for the detection of the periplasmic densities, including the collar, flanking bumps, and central stalk structures (Fig. 1). In stage III, upon receipt of an unknown signal, VirB4 recruits the spatially dynamic VirB11 ATPase, which in turn induces structural changes in the stalk and IMC (Fig. 4) of postulated importance for the transition from a pilus-generating machine to a substrate translocation channel (21, 52). Finally, in stage IV, upon substrate docking, the VirD4 substrate receptor binds VirB4, and the three ATPases coordinate substrate delivery through the lumen of the VirB4 hexamer and into the T4SS channel (37, 48). The proposed stage III and IV reactions are supported by our analyses of the Δ*traG_B11_* and Δ*traJ_D4_* mutant machines and recent findings for the Dot/Icm system (16, 21). Further *in situ* studies aimed at visualizing T4SSs with stably engaged VirB11 and VirD4 subunits, or of WT T4SSs in the act of translocating DNA or other substrates to recipient cells, will provide critical new information about structural transitions necessary for machine activation.

## MATERIALS AND METHODS

### Strains and growth conditions.

Bacterial strains, plasmids, and oligonucleotides used in this study are listed in Table S1 in the supplemental material. E. coli strains were grown at 37°C in Luria-Bertani (LB) agar or broth supplemented with the appropriate antibiotics (kanamycin at 100 μg ml^−1^, spectinomycin at 100 μg ml^−1^, and gentamicin at 10 μg ml^−1^). Minicells from E. coli strain UU2834 were used for all of the CryoET studies.

10.1128/mBio.02465-21.7 TABLE S1 List of strains, plasmids, and oligonucleotides used in these studies. Download Table S1, PDF file, 0.1 MB. Copyright © 2021 Khara et al. 2021 Khara et al. https://creativecommons.org/licenses/by/4.0/ This content is distributed under the terms of the Creative Commons Attribution 4.0 International license.

### Conjugation assays.

E. coli MG1655 strains carrying pKM101 or mutant variants were used as donors to transfer the plasmids into UU2834 recipients. Strains containing the pKM101 mutants also harbored a complementing plasmid. Cultures of donor and recipient cells grown overnight in the presence of the appropriate antibiotics at 37°C were diluted 1:1,000 in fresh LB medium and incubated with shaking for 1.5 h. When needed, cells were induced with arabinose (0.2% final concentration) and incubated with shaking for another 1.5 h. Equal volumes (50 μl) of donor and recipient cell cultures were mixed and incubated for 3 h at 37°C. Mating mixtures were serially diluted and plated onto LB agar containing antibiotics selective for transconjugants. Plasmid-carrying UU2834 strains were verified for the presence or absence of *tra* genes of interest by PCR. For matings to assess minicell donor capacity, minicells were spotted onto a nitrocellulose filter disc alone or with MC4100*rif^r^* recipient cells, and the mating mixes were incubated at 37°C for 1 h. Discs were suspended in LB medium, serially diluted, and plated onto LB agar plates containing the appropriate antibiotics selective for donors (to confirm the absence of viable donor cells), recipients, or transconjugants. Because minicells are nonviable, the frequency of transfer is reported as transconjugants per recipient. Matings were performed two times in triplicate, and results are presented as the mean frequencies of transfer with the standard errors of means (SEM).

### Isolation of minicells.

E. coli minicells were enriched essentially as described previously (56, 57). E. coli UU2834 cells harboring pKM101 or variants were grown overnight at 37°C in LB medium in the presence of spectinomycin and then subcultured (1:100) in fresh LB medium devoid of antibiotics at 37°C to an optical density at 600 nm (OD_600_) of 0.5. Anucleate minicells were selectively enriched by centrifugation at 2,000 × *g* for 10 min at room temperature to pellet rod-shaped cells. Next, the supernatant was centrifuged at 10,000 × *g* for 10 min to pellet the minicells. The minicells were resuspended in fresh LB medium and incubated at 37°C with gentle shaking for 45 min to reinitiate cell growth. Ceftriaxone (final concentration of 100 μg ml^−1^) was added to the minicell preparation to lyse growing cells, and the culture was further incubated at 37°C for 1 h. The preparation was centrifuged at 400 × *g* for 10 min to remove dead cells and debris. The supernatant was centrifuged at 10,000 × *g* for 10 min to harvest the minicells. The minicells were washed twice in fresh LB medium, filtered through a 0.45-μm filter (Millipore), and then used for the mating assay. To minimize possible breakage of the pKM101-encoded pilus and to concentrate minicells for CryoET analyses, UU2834 strains were grown overnight on LB agar plates at 37°C. Cells were gently scraped from the plate surface with an “L”-shaped rod and resuspended in phosphate-buffered saline (PBS). The cell suspension was centrifuged twice at 1,000 × *g* for 3 min to remove intact cells, the supernatant was then centrifuged at 10,000 × *g* for 20 min, and the minicell pellet was resuspended in PBS for the preparation of grids for CryoET.

### Preparation of frozen-hydrated specimens.

Minicells resuspended in PBS were mixed with 10-nm-diameter colloidal gold particles (Aurion bovine serum albumin [BSA] gold tracer, 10 nm) and deposited onto freshly glow-discharged, holey carbon grids (Quantifoil R2/1 200-mesh copper) for 1 min. After blotting the grids with filter paper, they were rapidly frozen in liquid ethane by using a gravity-driven plunger apparatus (58, 59).

### CryoET data collection and 3D reconstructions.

Frozen-hydrated specimens were imaged and data were processed using our previously established protocols (12, 28, 41). Briefly, specimens were subjected to imaging at −170°C using a Polara G2 electron microscope (FEI Company) equipped with a field emission gun and a direct detection device (Gatan K2 Summit). The microscope was operated at 300 kV at a magnification of ×15,000, resulting in an effective pixel size of 2.5 Å at the specimen level (17). The tomographic package SerialEM (60) was used to collect low-dose, single-axis tilt series in the dose fractionation mode with a defocus at ∼6 μm and a cumulative dose of ∼60 e^−^/Å^2^ distributed over 35 stacks. Each stack contains ∼8 images. Each tilt series was collected at angles from −51° to 51° with 3° fixed increments. We used Tomoauto (58) to expedite data processing, which included drift correction of dose-fractionated data using Motioncorr (61) and assembly of corrected sums into tilt series, automatic fiducial seed model generation, alignment and contrast transfer function correction of tilt series by IMOD (62), and reconstruction of tilt series into tomograms by Tomo3D (63). Each tomographic reconstruction was 3,710 by 3,838 by 2,400 pixels and ∼130 Gb in size.

### Subtomogram averaging and correspondence analysis.

The tomographic package I3 (64) was used for subtomogram analysis as described previously (65). A total of 837 T4SS_pKM101_ machines (400 by 400 by 400 voxels) were visually identified and then extracted from 1,781 cryo-tomographic reconstructions. Two of the three Euler angles of each T4SS_pKM101_ machine were estimated based on the orientation of each particle in the cell envelope. To accelerate image analysis, 4-by-4-by-4-binned subtomograms (100 by 100 by 100 voxels) were used for the initial alignment. The alignment proceeded iteratively, with each iteration consisting of three parts in which references and classification masks were generated, subtomograms were aligned and classified, and, finally, class averages were aligned to each other. At the initial iterations, a classification mask was applied to include the whole machine, and non-T4SS particles were sorted out and removed. For analysis of the IMC, a mask was applied to the IMC only; thus, the T4SS particles that did not show IMC density were sorted out, and the data set showing IMCs was used to further refine the IMC. Classification focusing on the OMCC displayed 14-fold symmetry; therefore, 14-fold symmetry was imposed in the following processing to assist in the initial alignment process. Classification focusing on the IMC showed a 6-fold symmetry feature, and in the following processing, 6-fold symmetry was imposed to assist in subtomogram alignments. After multiple cycles of alignment and classification for 4-by-4-by-4-binned subtomograms, 2-by-2-by-2-binned subtomograms were used for refinement. Fourier shell correlation (FSC) between the two independent reconstructions was used to estimate the resolution of the averaged structures (Fig. S2).

### 3D visualization.

IMOD was used to visualize the maps and generate 3D surface renderings of E. coli minicells. UCSF Chimera (66) (http://www.rbvi.ucsf.edu/chimera) was used to visualize subtomogram averages in 3D and for molecular modeling. The video clips for the movies in the supplemental material were made by using UCSF Chimera and further edited with iMovie.

### Data availability.

Density maps and coordinate data for the T4SS_pKM101_ machines determined by cryo-electron tomography have been deposited in the Electron Microscopy Data Bank (EMDB; https://www.ebi.ac.uk/emdb/) under identifiers EMD-24100 and EMD-24098. We declare that all other data supporting the findings of this study are available within the paper and its supplemental material.

10.1128/mBio.02465-21.1 TEXT S1 Supplemental references. Download Text S1, PDF file, 0.10 MB. Copyright © 2021 Khara et al. 2021 Khara et al. https://creativecommons.org/licenses/by/4.0/ This content is distributed under the terms of the Creative Commons Attribution 4.0 International license.

10.1128/mBio.02465-21.9 MOVIE S2 3D visualization of the T4SS_pKM101_ showing architectural features of the OMCC and IMC and its comparison with the purified VirB_3-10_ complex from R388. Download Movie S2, MOV file, 3.8 MB. Copyright © 2021 Khara et al. 2021 Khara et al. https://creativecommons.org/licenses/by/4.0/ This content is distributed under the terms of the Creative Commons Attribution 4.0 International license.
