# Supplementary material for: In Situ Visualization of the pKM101-Encoded Type IV Secretion System Reveals a Highly Symmetric ATPase Energy Center
Source: mBio. 2021 Oct 12;12(5):e02465-21. doi: 10.1128/mBio.02465-21 (PMC8510550; doi:10.1128/mBio.02465-21)
Supplement: FIG S1 [file mbio.02465-21-sf001.pdf]

A

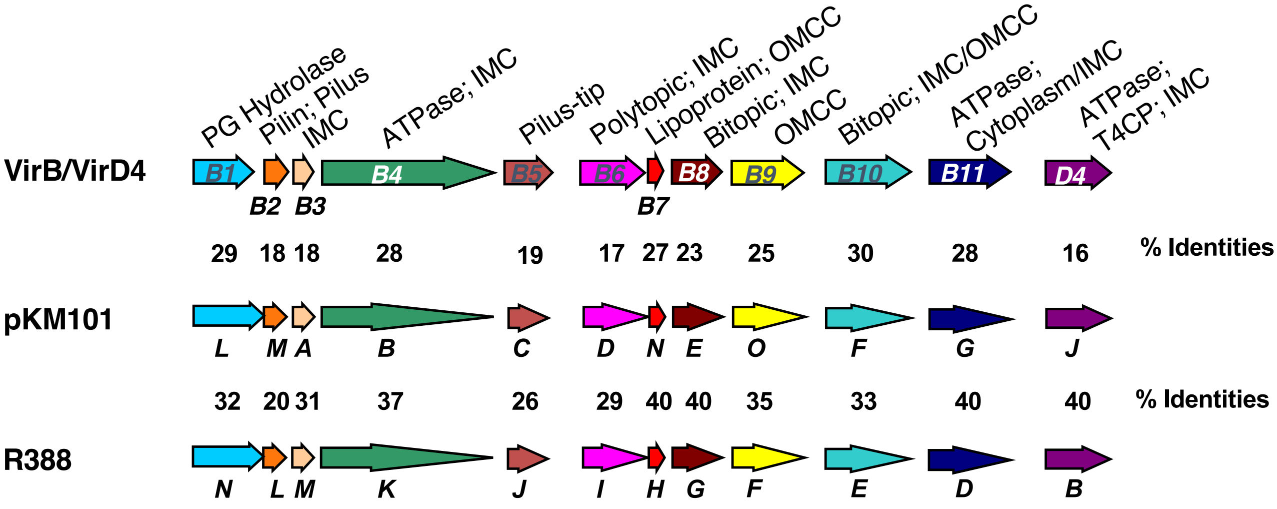


B

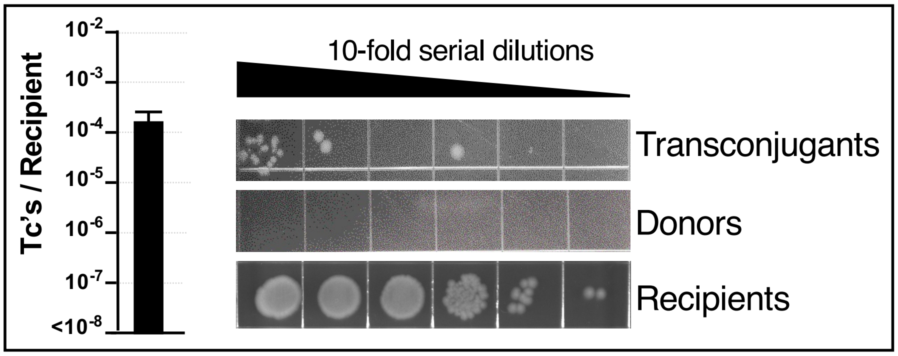


**Fig. S1.** Genes and encoded functions of "minimized" type IV secretion systems (T4SSs) in Gram-negative species and determination of donor potential of minicells. **(A)** The *Agrobacterium tumefaciens* VirB/VirD4 system is assembled from 11 VirB subunits and the VirD4 substrate receptor (4). Homologs in the pKM101 and R388 systems are color-matched; letters refer to the *tra* or *trw* names, e.g., *traL, trwN,* for the pKM101- or R388-encoded genes. Percent identities of proteins relative to the reference pKM101 proteins are shown. Percent identities were determined using Multalin (5). **(B)** Minicells from *E. coli* UU2834(pKM101) conjugatively transfer pKM101 to viable recipient cells. Purified minicells were mated with recipient cells for 1 h on LB plates. Transfer frequencies are reported as transconjugants (Tc's) / recipient cell. Five microliters of serially diluted mating mixtures were spotted onto plates selective for growth of transconjugants, viable donors, and recipients. Mating mixtures were devoid of viable donor cells, establishing that minicells are responsible for delivering pKM101 to recipients.
