# Supplementary material for: In Situ Visualization of the pKM101-Encoded Type IV Secretion System Reveals a Highly Symmetric ATPase Energy Center
Source: mBio. 2021 Oct 12;12(5):e02465-21. doi: 10.1128/mBio.02465-21 (PMC8510550; doi:10.1128/mBio.02465-21)
Supplement: FIG S2 [file mbio.02465-21-sf002.pdf]

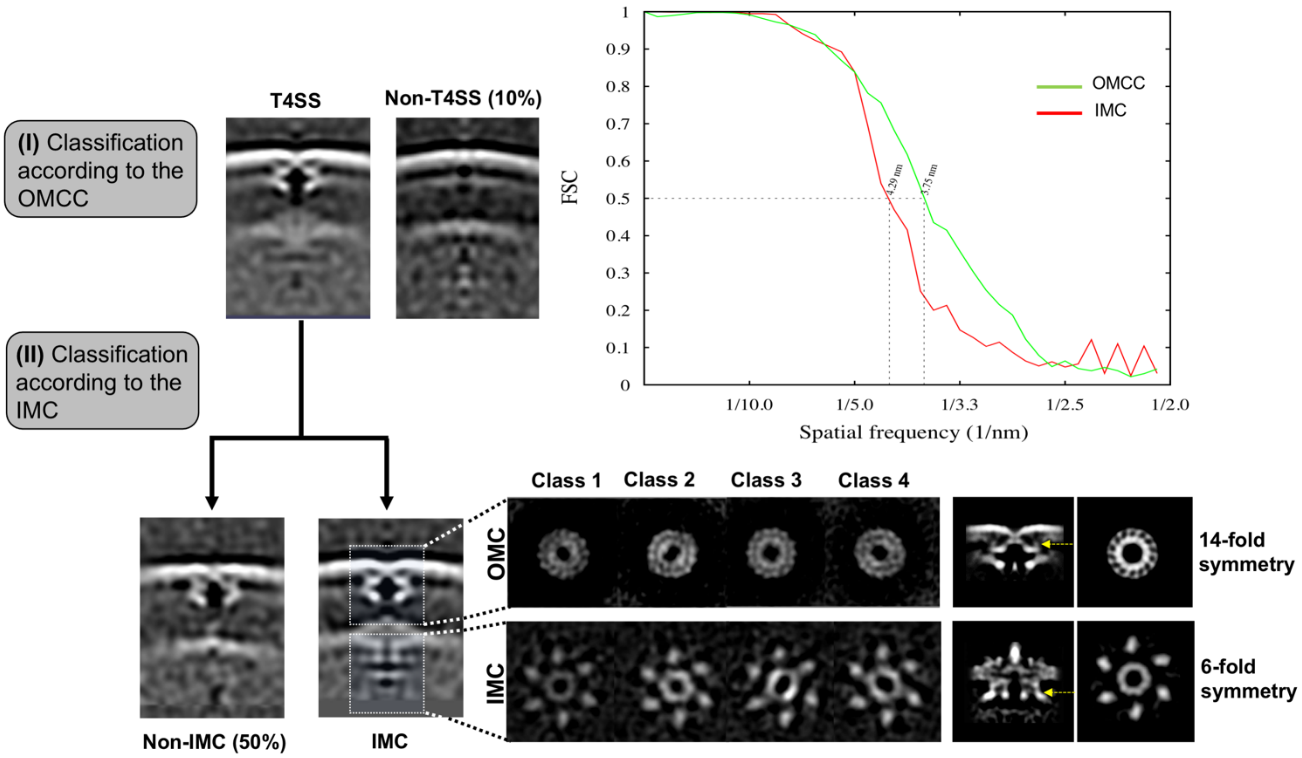

Fig. S2. Workflow for *in situ* CryoET. Approach taken to solve the *in situ* structure of type IV secretion system encoded by plasmid pKM101 ($T4SS_{pKM101}$). Machines identified initially by the presence of the readily visualized outer membrane core complex (OMCC) were further analyzed, and initially picked portions of the cell envelope that on further analysis clearly lacked the OMCC were discarded. Of the T4SS complexes with clearly detectable OMCCs, about half lacked discernible densities corresponding to the IMC and were discarded. We propose that these correspond to assembly intermediates on the basis of early findings that T4SSs assemble in stepwise fashion, first building intrinsically stable OMCCs and then the less stable IMCs (see (4). The OMCC and IMC subassemblies of the remaining T4SS complexes were aligned and subjected to multivariate statistical analysis and hierarchical ascendant classification to generate four class averages. The 14- and 6-fold symmetrical features of the OMCC and IMC subassemblies were readily evident in the initial classifications. During refinements of these subassemblies, 14- and 6-fold symmetries were imposed, yielding the 3D averages presented at the far right in cross-section and end-on views of the regions marked with yellow arrows. Top right: Fourier shell correlation plots of sub-volume averages aligned on the OMCC (green) and IMC (red) regions.
