# Supplementary material for: In Situ Visualization of the pKM101-Encoded Type IV Secretion System Reveals a Highly Symmetric ATPase Energy Center
Source: mBio. 2021 Oct 12;12(5):e02465-21. doi: 10.1128/mBio.02465-21 (PMC8510550; doi:10.1128/mBio.02465-21)
Supplement: FIG S3 [file mbio.02465-21-sf003.pdf]

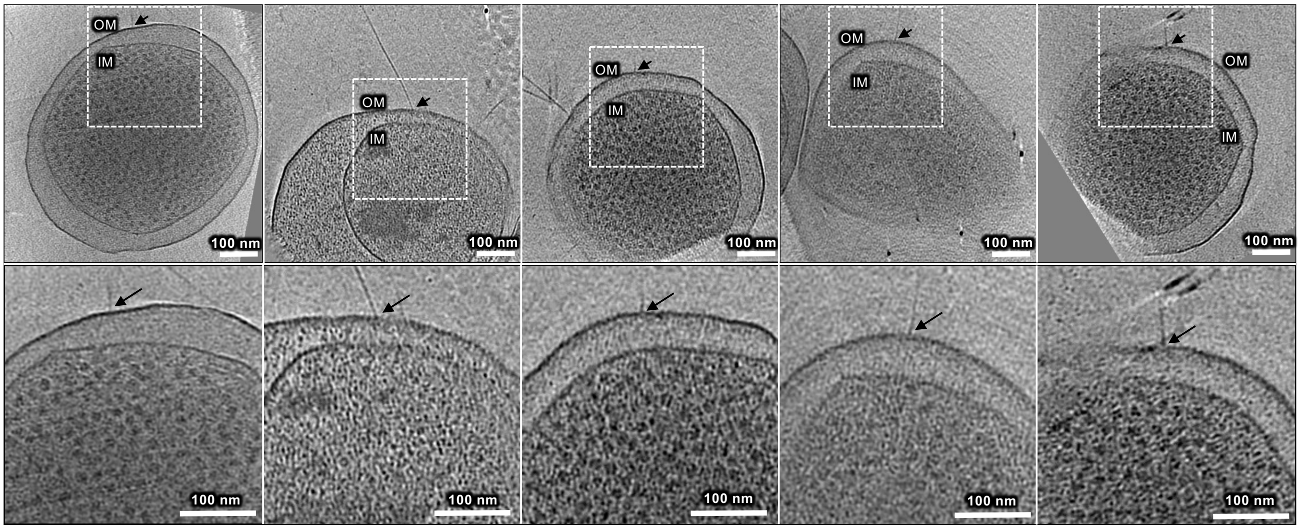

Fig. S3. Detection of pKM101 pilus docked on *E. coli* outer membrane. Docked pili (indicated by black arrow) lack detectable associated densities in the OM or periplasm. The boxed regions were magnified and shown in the lower panels.
