# Supplementary material for: In Situ Visualization of the pKM101-Encoded Type IV Secretion System Reveals a Highly Symmetric ATPase Energy Center
Source: mBio. 2021 Oct 12;12(5):e02465-21. doi: 10.1128/mBio.02465-21 (PMC8510550; doi:10.1128/mBio.02465-21)
Supplement: FIG S4 [file mbio.02465-21-sf004.pdf]

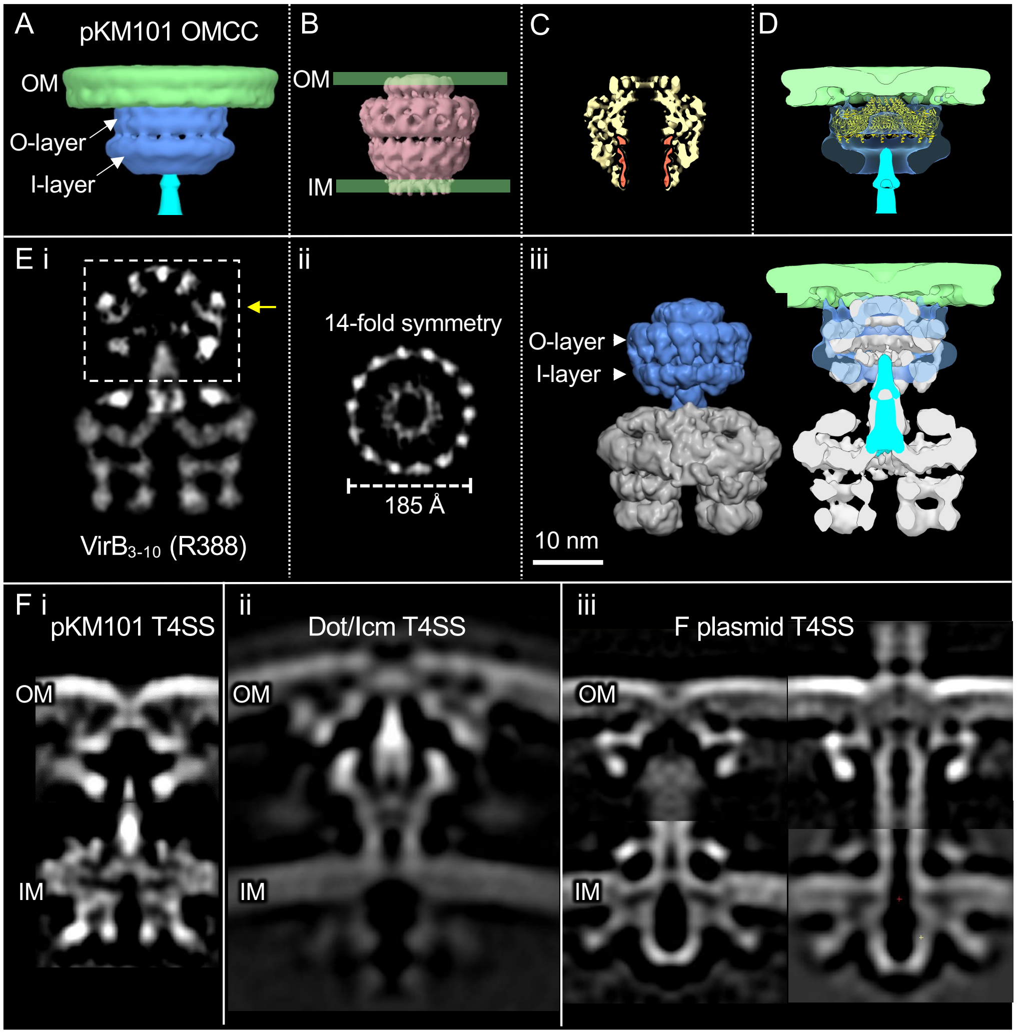

Fig. S4. Comparisons of the outer membrane core complexes (OMCCs) and stalks/cylinders of 'minimized' and 'expanded' T4SSs. **(A)** OMCC of the pKM101 T4SS visualized in this study by *in situ* CryoET. **(B, C)** Structures obtained by overproduction and purification of the pKM101 OMCC followed by characterization with single particle cryoelectron microscopy (CryoEM) (**B**, EMD:5031, **C**, EMD:2232) (6, 7). The red lines in panel C image denote the $TraF_{B10}$ linker region, which extends from the O-layer of the OMCC through the I-layer to the inner membrane (not shown) **(D)** The *in situ* $OMCC_{pKM101}$ with a superimposed atomic structure of the O-layer obtained by X-ray crystallography (PDB:3JQO) (8). **(E i)** A central slice of the averaged structure of the $VirB_{3-10}$ complex obtained by overproduction of the R388-encoded VirB3 - VirB10 homologs followed by affinity purification and analysis by negative-stain electron microscopy (EMD:2567) (9). **(Eii)** A cross-section view of the OMCC at the position marked by a yellow arrow in panel **Ei** showing 14-fold symmetry. **(Eiii)** The $VirB_{3-10}$ structure with the superimposed OMCC from the $T4SS_{pKM101}$ visualized by *in situ* CryoET. **(Fi-iii)** Comparison of the central stalk/cylinder of the 'minimized' (pKM101) and 'expanded' Dot/Icm and F plasmid-encoded T4SSs visualized by *in situ* CryoET.
