# Supplementary material for: In Situ Visualization of the pKM101-Encoded Type IV Secretion System Reveals a Highly Symmetric ATPase Energy Center
Source: mBio. 2021 Oct 12;12(5):e02465-21. doi: 10.1128/mBio.02465-21 (PMC8510550; doi:10.1128/mBio.02465-21)
Supplement: FIG S5 [file mbio.02465-21-sf005.pdf]

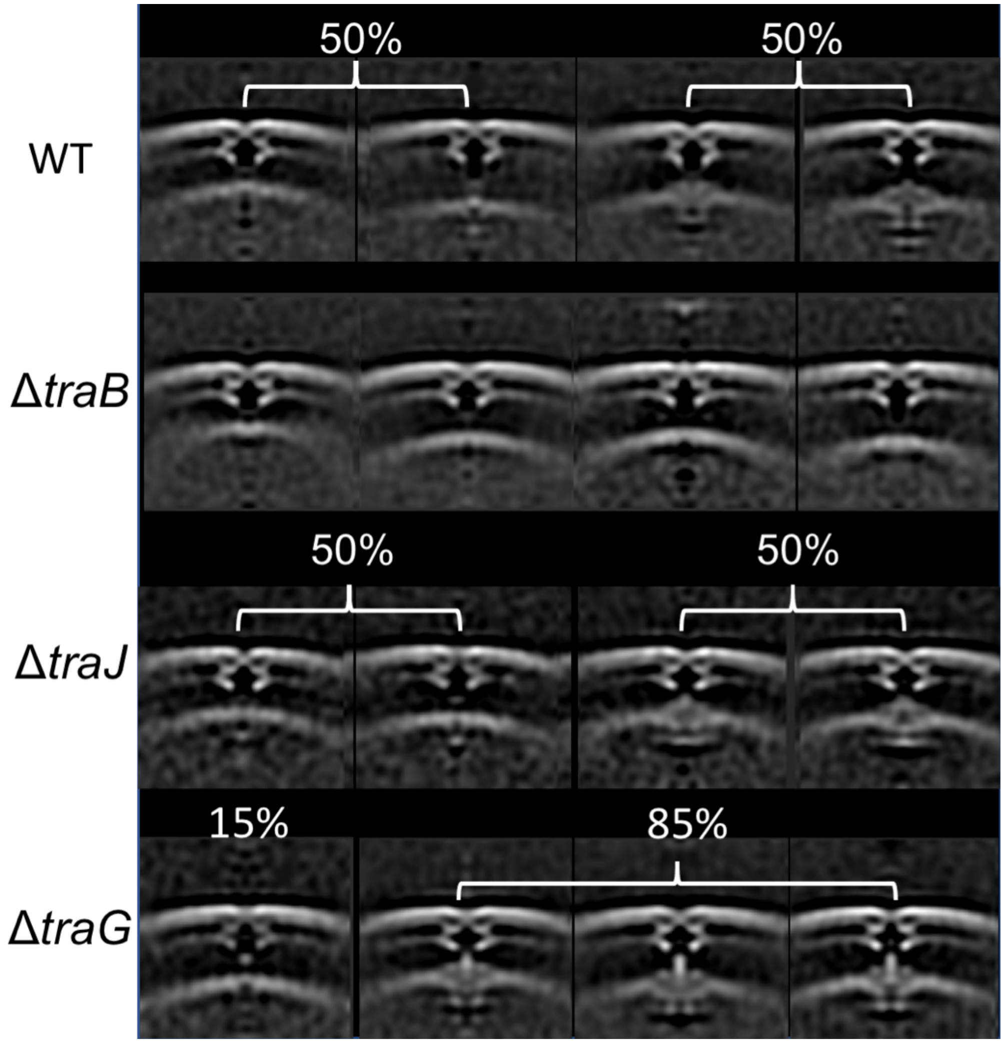

Fig. S5. Heterogeneity of the pKM101 T4SS machines revealed by class sub-volume averaging. For the T4SS structures from each strain, four representative class averages are shown. With minicells from strain UU2834(pKM101), the OMCC was detected in association with the IMC in about one-half and without a discernible IMC in the other half of the visualized machines. Only machines composed of both the OMCC and IMC were used to generate the refined structure. The Δ*traB* mutant machines invariably were composed of the OMCC without discernible IMC densities. The Δ*traJ* mutant machines appeared similar to the WT machines, insofar as half were associated with and the other half devoid of detectable IMC densities; only mutant machines bearing a discernible IMC were used to generate the refined structure. The Δ*traG* mutant machines were of interest because only ~15 % were composed of an OMCC without an associated IMC, and among the remaining 85 % the associated IMCs were even better defined than the WT machines particularly with respect to densities corresponding to the periplasmic collar and stalk and cytoplasmic complex; these latter machines were used to generate the refined structure.
