# Supplementary material for: In Situ Visualization of the pKM101-Encoded Type IV Secretion System Reveals a Highly Symmetric ATPase Energy Center
Source: mBio. 2021 Oct 12;12(5):e02465-21. doi: 10.1128/mBio.02465-21 (PMC8510550; doi:10.1128/mBio.02465-21)
Supplement: TABLE S1 [file mbio.02465-21-st001.pdf]

Supplementary Information for:

In Situ Visualization of the pKM101-Encoded Type IV Secretion System Reveals a Highly Symmetric ATPase Energy Center at the Channel Entrance

Pratick Khara, Liqiang Song, Peter J. Christie*, Bo Hu*

Department of Microbiology and Molecular Genetics, McGovern Medical School,

6431 Fannin St, Houston, Texas 77030

*Correspondence to: Bo Hu or Peter J. Christie

Department of Microbiology and Molecular Genetics, McGovern Medical School, Houston, TX 77030.

Fax: 713-500-5499

Hu:
Phone: 713-500-5891
e-mail: Bo.Hu@uth.tmc.edu

Christie:
Phone: 713-500-5440
e-mail: Peter.J.Christie@uth.tmc.edu

This PDF file includes:
Table S1
Figures S1-S6
References

Other supplementary materials for this manuscript include the following:
Movies S1, S2

Table S1. List of strains, plasmids, and oligonucleotides used in these studies.

Strains	Genotype	Source
UU2834	*(tsr)5547 (aer)1 ygjG::genR (trg)4543 min::kan (flbBflaH)4 mreB-A125V thr(Am)-1 leuB6 his-4 metF(Am)159 rpsL136 [thi-1 ara-14 lacY1 mtl-1 xyl-5 tonA31 tsx-78]*	(1)
MG1655	*ilvG rpb-50 rph-1 ΔlacU169*	*E. coli* Genetic Stock Center
MC4100*rif*^{R}	F- *[araD139]*$_{B/r}$ Δ(*argF-lac*)*169* λ^{-} *e14*$^{-}$ *flhD5301* Δ(*fruK-yeiR*)*725*(*fruA25*) *relA1 rpsL150*(*strR*) *rbsR22* Δ(*fimB-fimE*)*632(::IS1) deoC1 rifR*	Lab Stock

Plasmids	Characteristics	
pKM101-Spc^{r}	Spc^{r}; pKM101 derivative	(2)
pJG1004	Spc^{r}; pKM101-Spc^{r} with a Δ*traB* mutation	(3)
pJG1011	Spc^{r}; pKM101-Spc^{r} with a Δ*traG* mutation	(3)
pKM101-Spc^{r}-Δ*traJ*	Spc^{r}; pKM101-Spc^{r} with a Δ*traJ* mutation	(2)
pMS4	Kan^{r}; pBAD24Kan^{r} with P$_{BAD}$::*traB*$_{pKM101}$	(3)
pMS11	Kan^{r}; pBAD24Kan^{r} with P$_{BAD}$::*traG*$_{pKM101}$	(3)
pTB26	Kan^{r}; pBAD24Kan^{r} with P$_{BAD}$::*traJ*$_{pKM101}$	(2)

Oligonucleotides	Sequences (5′ to 3′)	Purpose
pKM_up_traB-F	ATTATGGGCCTGTGGTTGAAAACAAATTCAG	Confirmation of Δ*traB* mutation in UU2834
pKM_dn_traB-R	TTCACCCTGTTTAATCAGCTCGGTGGG	Confirmation of Δ*traB* mutation in UU2834
pKM_mid_traB-F	GCCAGGCCAGCTTAACATGCTTAAAGAAG	Confirmation of Δ*traB* mutation in UU2834
pKM_mid_traB-R	CACAAATGCGGAACAGGTTTTTAATGAGGG	Confirmation of Δ*traB* mutation in UU2834
pKM_up_traG-F	CTCTACGATCAGCAGGGTGATGCG	Confirmation of Δ*traG* mutation in UU2834

pKM_dn_traG-R	CTTCAGGCGAGTAGCCAACCTGAAC	Confirmation of Δ*traG* mutation in UU2834
pKM_mid_traG-F	GCAGATTTTATTAGGAACCTTGCTAAATCGTTGG	Confirmation of Δ*traG* mutation in UU2834
pKM_mid_traG-R	ATAATCCCACGCAGCATCATCCCTAAG	Confirmation of Δ*traG* mutation in UU2834
pKM_up_traJ-F	CCGGCAGGACAGATGATGTTGCATG	Confirmation of Δ*traJ* mutation in UU2834
pKM_dn_traJ-R	CAAGCAGCTCTTTAAACCGGGCTG	Confirmation of Δ*traJ* mutation in UU2834
pKM_mid_traJ-F	CCATACCTACATACGCTGAGAACTTGC	Confirmation of Δ*traJ* mutation in UU2834
pKM_mid_traJ-R	CATGTTAGCCAGAATGGTCTGAGCC	Confirmation of Δ*traJ* mutation in UU2834
