# Supplementary material for: In Situ Visualization of the pKM101-Encoded Type IV Secretion System Reveals a Highly Symmetric ATPase Energy Center
Source: mBio. 2021 Oct 12;12(5):e02465-21. doi: 10.1128/mBio.02465-21 (PMC8510550; doi:10.1128/mBio.02465-21)
Supplement: TEXT S1 [file mbio.02465-21-s0001.pdf]

Movie S1. 3-D visualization of a tomographic reconstruction and the $T4SS_{pKM101}$ in *E. coli* minicells.

Movie S2. 3-D visualization of the $T4SS_{pKM101}$ showing architectural features of the OMCC and IMC and its comparison with purified $VirB_{3-10}$ complex from R388.

References

1. **Hu B, Khara P, Christie PJ**. 2019. Structural bases for F plasmid conjugation and F pilus biogenesis in *Escherichia coli*. Proc Natl Acad Sci U S A **116:**14222-14227.
2. **Whitaker N, Berry TM, Rosenthal N, Gordon JE, Gonzalez-Rivera C, Sheehan KB, Truchan HK, VieBrock L, Newton IL, Carlyon JA, Christie PJ.** 2016. Chimeric coupling proteins mediate transfer of heterologous type IV effectors through the *Escherichia coli* pKM101-encoded conjugation machine. J Bacteriol **198:**2701-2718.
3. **Gordon JE, Costa TRD, Patel RS, Gonzalez-Rivera C, Sarkar MK, Orlova EV, Waksman G, Christie PJ**. 2017. Use of chimeric type IV secretion systems to define contributions of outer membrane subassemblies for contact-dependent translocation. Mol Microbiol **105:**273-293.
4. **Christie PJ, Atmakuri K, Krishnamoorthy V, Jakubowski S, Cascales E. 2005.** Biogenesis, architecture, and function of bacterial type IV secretion systems. Annu Rev Microbiol **59:**451-485.
5. **Corpet F.** 1988. Multiple sequence alignment with hierarchical clustering. Nucleic Acids Res **16:**10881-10890.
6. **Fronzes R, Schafer E, Wang L, Saibil HR, Orlova EV, Waksman G.** 2009. Structure of a type IV secretion system core complex. Science **323:**266-8.
7. **Rivera-Calzada A, Fronzes R, Savva CG, Chandran V, Lian PW, Laeremans T, Pardon E, Steyaert J, Remaut H, Waksman G, Orlova EV**. 2013. Structure of a bacterial type IV secretion core complex at subnanometre resolution. EMBO J **32:**1195-204.
8. **Chandran V, Fronzes R, Duquerroy S, Cronin N, Navaza J, Waksman G.** 2009. Structure of the outer membrane complex of a type IV secretion system. Nature **462:**1011-1015.
9. **Low HH, Gubellini F, Rivera-Calzada A, Braun N, Connery S, Dujeancourt A, Lu F, Redzej A, Fronzes R, Orlova EV, Waksman G.** 2014. Structure of a type IV secretion system. Nature 508:550-553.
